# Early Diagnosis and Treatment Outcomes of Fournier Gangrene in a Tertiary Center

**DOI:** 10.7759/cureus.63815

**Published:** 2024-07-04

**Authors:** Ahmed Alasker, Rayan W Almasari, Abdullah Alhaidar, Abdulaziz F Alajmi, Rayan K Alsaleh, Ammar Aloufi

**Affiliations:** 1 Division of Urology, King Abdulaziz Medical City Riyadh, Riyadh, SAU; 2 Medicine, King Abdullah International Medical Research Center, Riyadh, SAU; 3 Collage of Medicine, King Saud Bin Abdulaziz University for Health Sciences, Riyadh, SAU; 4 College of Medicine, King Saud Bin Abdulaziz University for Health Sciences, Riyadh, SAU

**Keywords:** urological infections, mortality rate, urological emergencies, necrotizing fasciitis, fournier gangrene

## Abstract

Introduction: Fournier gangrene is an uncommon urological emergency caused by microbial agents, resulting in necrosis of the genitalia and perineum. This study aims to evaluate the outcomes of early diagnosis and management of Fournier gangrene at KAMC in Riyadh, Saudi Arabia.

Methods: A retrospective cohort study was conducted at KAMC, Saudi Arabia. The study population included all adult patients diagnosed with Fournier gangrene between 2015 and 2022. Data analysis was performed using RStudio (RStudio, Boston, MA). Frequencies and percentages were used to present categorical data, while medians and interquartile ranges were used to express numerical variables.

Results: The study included 41 patients with Fournier gangrene, the majority (95.12%) being male with a median age of 60 years. The most prevalent comorbidity was diabetes mellitus (85.37%). Ten patients presented to the hospital with sepsis, two of whom were in shock. Within 90 days of admission, two of them had expired. This resulted in a 20% mortality rate among septic patients. The mean FGSI in patients who had died during hospital stays was approximately two times the mean in surviving patients (8.17 and 4.32, respectively). The most utilized imaging study was a CT scan (70.7%). Most patients had undergone multiple debridements (87.7%). The median number of debridements per patient was three, and the interval between each debridement was three days. The most frequent tissue culture finding was mixed organisms, followed by *Escherichia coli*. Regarding empiric antibiotics, tazocin was the most used, accounting for 22.0%. The most frequently performed adjunctive procedure was the placement of a suprapubic catheter, accounting for 41.5%. Roughly 43.90% required a blood transfusion. Within 90 days of admission, six patients had died, which makes the mortality rate 14.6%. Four of them had died within 30 days of admission (9.76%).

Conclusion: Fournier gangrene is a surgical emergency that requires prompt attention and resuscitation, antibiotic therapy, and surgical debridement. The study identified the demographic factors of patients who presented with the disease and provided the incidence, mortality rate, and outcomes of the disease. It also identified specifics of the pharmacological and surgical management and hospital courses.

## Introduction

Fournier gangrene is an uncommon yet fatal necrotizing infection of the perineum and genitalia. Numerous microorganisms, most frequently anaerobic bacteria, are responsible for its development. Early diagnosis and intensive treatment are required to avoid serious consequences. Such as sepsis, multi-organ failure, and death [1,2].

Typical bacteria found in the perineum and genitalia, such as *Escherichia coli*, *Klebsiella pneumoniae*, *Bacteroides fragilis*, and *Staphylococcus aureus*, were reported to be the most frequently contributing microorganisms to the poly-microbial presentation in individuals with Fournier gangrene [3]. Trauma to perineal areas has been described as a potential cause of infection. Risk factors for Fournier gangrene include diabetes mellitus, long-term steroid use, human immunodeficiency virus (HIV), lymphoproliferative disorders, and cytotoxic drug use [4]. Furthermore, clinical indicators such as fluctuance, crepitus, local pain, and lesions on the genitalia and perineum are the mainstays of the Fournier gangrene diagnosis [5]. The most common non-specific laboratory findings in Fournier gangrene are anemia, leukocytosis, thrombocytopenia, and abnormalities of electrolytes [5].

Two clinical scoring systems are used to evaluate the severity of Fournier gangrene, which are Fournier Gangrene Severity Index (FGSI) and the Laboratory Risk Indicator for Necrotizing Fasciitis (LRINEC) [6]. Imaging confirms the diagnosis, which shows air that represents infection. Standard imaging is X-ray; however, CT is the gold standard [6]. Effectively treating Fournier gangrene is difficult [7]. This is a result of the necrosis, quick progression, and systemic manifestations that lead to sepsis and shock [7].

This study aims to evaluate the outcomes of early diagnosis and management of Fournier gangrene, specifically studying the incidence, mortality rate, risk factors, and hospital stay at King Abdulaziz Medical City (KAMC) in Riyadh, Saudi Arabia.

This article was previously presented as an oral abstract presentation at the 35th Saudi Urological Association Conference on February 15, 2024.

## Materials and methods

This was a retrospective cohort study conducted at King Abdulaziz Medical City in Riyadh, Saudi Arabia. The study included all adult patients diagnosed with Fournier gangrene between the years 2015 and 2022. Patients with other perineal manifestations that were not diagnosed as Fournier gangrene were excluded from the study.

The primary outcome of the study was to determine the incidence and quantify the mortality rate of Fournier gangrene at KAMC. Secondary outcomes included length of hospital stay and identification of the risk factors for Fournier gangrene.

Data were collected from the BestCare system using a Google spreadsheet. Variables like age, weight, risk factors, diagnostic and treatment methods, microbial agents, and postoperative outcomes were collected. The data were cleaned and reviewed to check for accuracy and mistakes.

Statistical analysis was performed using RStudio (R version 4.3.0, RStudio, Boston, MA). Frequencies and percentages were used to present categorical data, while median and interquartile range (IQR) were used to express numerical variables. Bar charts and pie charts were used to present frequency data.

## Results

Demographic characteristics and clinical history

The study included a total of 41 patients with Fournier gangrene, with a majority of 39 (95.1%) being male. The median age of the patients was 60.0 years (interquartile range: 51.0-67.0). The most prevalent comorbidity observed among the patients was diabetes mellitus (DM) in 35 patients (85.37%). Other notable comorbidities included hypertension in 23 patients (56.10%). Cardiac disease in 19 patients (46.34%), renal disease in 13 patients (31.71%), chronic limb ischemia in 12 patients (29.27%), and dyslipidemia in 12 patients (29.27%). Smoking was reported by 10 patients (24.39%). The frequencies and percentages of other risk factors, such as a history of radiation, bed sores, chronic Foley's, suprapubic catheter, malignancy, anti-neoplastic medication usage, substance abuse, and trauma, were relatively low, ranging from 2.4% to 14.63% (Tables 1-2).

**Table 1:** Demographic characteristics BMI: body mass index

Parameter	Interquartile range	Mean	Frequency	Percentage
Age (years)	51–67	60		
Gender				
Male			39	95.10%
Female			2	4.90%
Height (cm)	162–173	168		
Weight (kg)	70–93	80		
BMI (kg/m^2^)	25.6–31.5	27.9		

**Table 2:** Comorbidities

Parameter	Frequency	Percentage
﻿﻿﻿Diabetes mellitus	35	85.37%
﻿﻿﻿Hypertension	23	56.10%
﻿﻿﻿Cardiac disease	19	46.34%
﻿﻿﻿Renal disease	13	31.71%
Chronic limb ischemia	12	29.27%
﻿﻿﻿Dyslipidemia	12	29.27%
﻿﻿﻿Smoking	10	24.39%
﻿﻿﻿Chronic transurethral catheterization	6	14.63%
﻿﻿﻿Malignancy	4	9.76%
﻿﻿﻿﻿Bed sores	4	9.76%
﻿﻿﻿﻿History of radiation	3	7.32%
﻿﻿﻿﻿Suprapubic catheter at admission	3	7.32%
﻿﻿﻿﻿Alcohol	2	4.88%
Anti-neoplastic medications	2	4.88%
﻿﻿﻿﻿Long-term steroid use	1	2.44%
﻿﻿﻿﻿Substance abuse	1	2.44%
﻿﻿﻿﻿Trauma	1	2.44%

Anatomical extent of the condition and the presenting signs and symptoms

**Figure 1:**
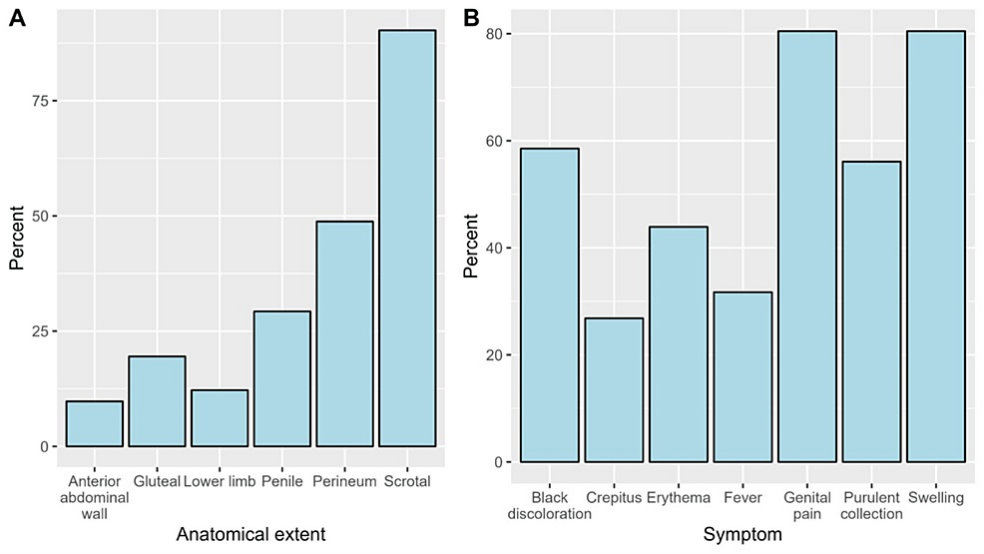
The proportions of the anatomical extent of the disease (A) and symptoms at presentation (B)

Regarding the anatomical extent of the disease, a clinical anatomical extension of the disease involved the scrotum among most patients (90%), followed by the perineum (49%) and the penis (29%) (Figure 1A). The most reported symptoms were genital pain (80.5%), swelling (80.5%), and black discoloration (58.5%) (Figure 1B). The most common infectious state upon presentation was a localized infection, observed in 31 patients (75.6%). Sepsis was seen in eight patients (19.5%) on admission. Septic shock was observed in two patients (4.9%) upon presentation. The median duration of the disease prior to presentation was 5.5 days (interquartile range: 3.0-10.0). In terms of imaging investigations, computed tomography (CT) was the most commonly utilized modality, performed in 29 patients (70.7%). X-ray imaging was performed in 10 patients (24.4%), ultrasound in 9 patients (22.0%), and magnetic resonance imaging (MRI) in only 1 patient (2.4%, Table 3). The Fournier Gangrene Severity Index was calculated. Patients who survived 90 days since admission scored significantly lower than patients who have died. The mean FGSI among living patients was 4.32. The mean FGSI among dead patients was 8.17 (Table 4).

**Table 3:** Presentation and diagnostics CT: computed tomography scan, MRI: magnetic resonance imaging, US: ultrasound

Parameter	Frequency	Percentage
Mode of presentation		
Localized infection	31	75.6%
Sepsis	8	19.5%
Septic shock	2	4.9%
Confirmatory imaging		
None	6	14.6%
X-ray	10	24.4%
CT	29	70.7%
MRI	1	2.4%
US	9	22%

**Table 4:** Fournier Gangrene Severity Index (FGSI)

Mortality status	Mean
Total	4.56
Living	4.32
Dead	8.17

Characteristics of surgeries

The surgeries performed for Fournier gangrene had certain characteristics within the studied population. The times of debridements conducted for Fournier gangrene varied, with a median of 3.0 (interquartile range: 2.0-9.5) debridements per patient. The median time between operations ranged between patients, with a median of 3.0 days (interquartile range: 1.5-7.5). Multiple debridements were performed in 87.80% of the cases. Regarding the surgically explored areas, the scrotum was the most frequently explored region, occurring in 92.68% of cases. The perineum was the next most common area explored, observed in 60.98% of cases. Other explored areas included the abdomen (21.95%), penis (17.07%), and perianal area (14.63%, Table 5).

**Table 5:** Surgically explored areas

Area	Frequency	Percentage
Scrotum	39	95.12%
Perineum	25	60.98%
Abdomen	9	21.95%
Penis	7	17.07%
Perianal area	6	14.63%
Gastrointestinal	4	9.76%
Gluteal region	2	4.88%
Vulva	1	2.44%
Thigh	1	2.44%
Suprapubic area	1	2.44%

**Figure 2:**
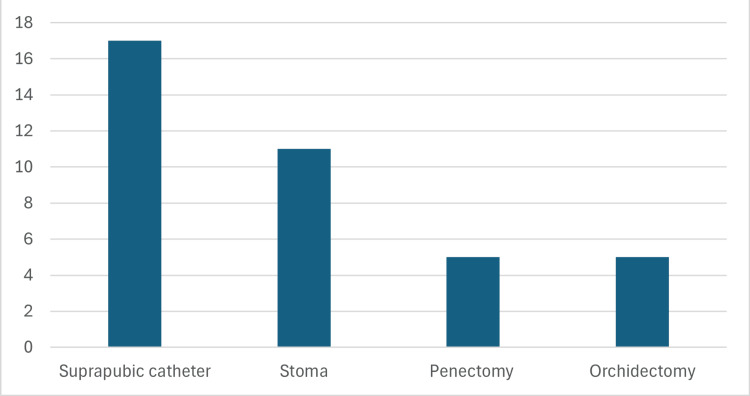
Frequency of adjunctive procedures performed in the study population

The most frequently performed adjunctive procedure was the placement of a suprapubic catheter, performed in 17 patients (41.5%). Stoma creation was the next most common procedure, performed in 11 patients (26.8%). Penectomy and orchidectomy were each performed in five patients (12.2%, Figure 2).

Outcomes of surgeries

**Figure 3:**
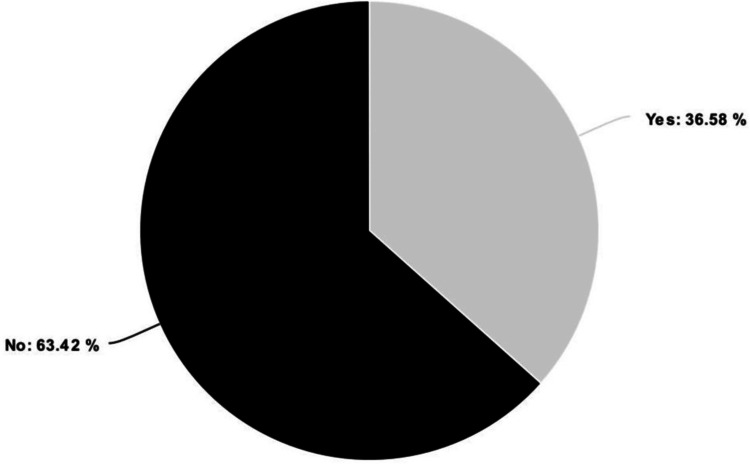
The proportions of patients admitted to ICU

**Figure 4:**
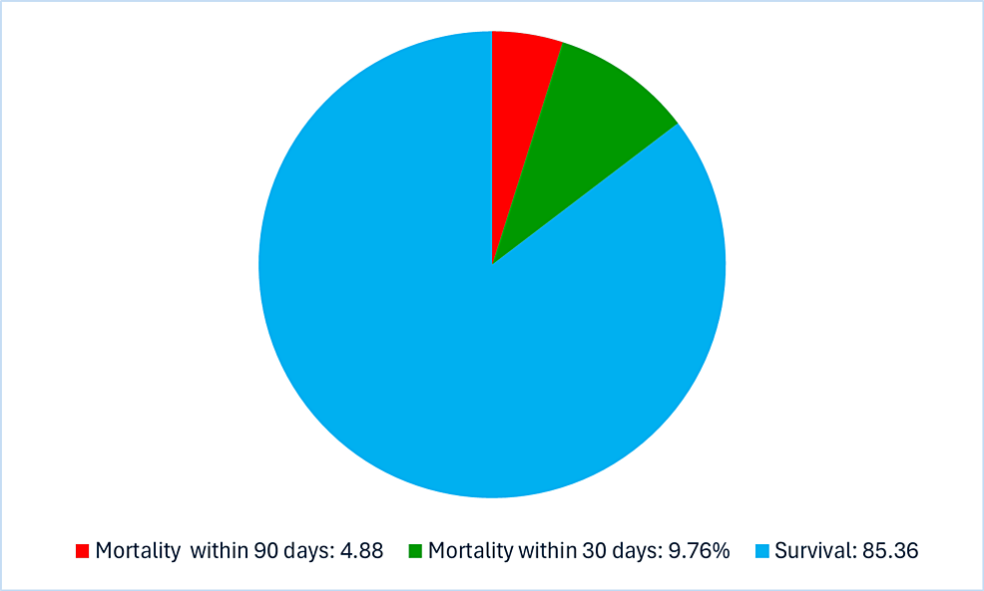
The proportions of 30-day and 90-day mortality

A total of 18 patients (43.9%) required blood transfusions, and the median number of transfused units was 2.0 units (1.3 to 6.0). Out of the total study population, 15 patients were admitted to the intensive care unit (ICU, Figure 3). The median duration of the ICU stay was eight days (interquartile range: 4-17). The reasons for ICU admission varied among the patients. The most common cause was septic shock, accounting for 53.3% of the admitted ICU cases. Overall, renal failure was observed in 7.3% of the cases. Pneumonia occurred in 4.9% of the patients, while other postoperative complications accounted for around 7.3% (Table 6). Within 90 days of admission, six patients had died, which makes the mortality rate 14.6%. Four of them had died within 30 days of admission (9.76%) (Figure 4). Out of the ten patients who presented to the hospital with sepsis and septic shock, two had died. This results in a mortality rate of 20% for septic presentations.

**Table 6:** Outcomes of surgery

Parameter	Mean	Interquartile range	Frequency	Percentage
Duration of hospital admission (days)	27	12–40.5		
Patients admitted to ICU			15	36.58%
Duration of ICU admission (days)	8	4–17		
Cause of ICU admission				
Septic shock			8	53.30%
Post-op observation			3	20%
Hemorrhagic shock			1	6.66%
Cardiac arrest			1	6.66%
Hypoxemia			1	6.66%
Atrial fibrillation			1	6.66%
Postoperative complications				
Pneumonia			2	4.9%
Renal failure			3	7.3%

Outcomes of the microbiological testing and patterns of antibiotic prescription

**Figure 5:**
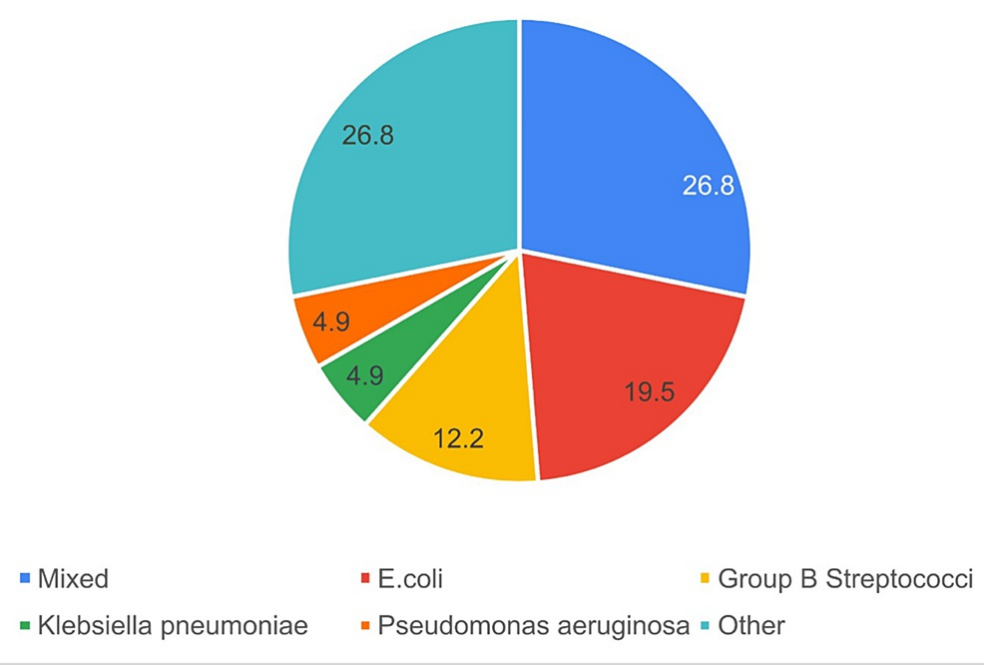
The proportions of the isolated microorganisms

The majority of debrided tissue cultures (92.7%) were positive, indicating the presence of microorganisms. Among the positive culture results, various organisms were isolated. However, the most frequent results were mixed organisms (26.8%), followed by *E. coli* (19.5%, Figure 5). Regarding the empiric antibiotics used in the treatment of Fournier Gangrene, various antibiotics were administered to the patients. The most frequently used empirical antibiotic was Piperacillin/tazobactam, accounting for 62.29% of cases, followed by Vancomycin (43.90%), and Meropenem (39%, Table 7). Tailored antibiotic agents used after culture results were mostly Piperacillin/tazobactam (47.37%) and Meropenem (47.37%, Table 8).

**Table 7:** Empirical antibiotics

Antibiotic	Frequency	Percentage
Piperacillin/tazobactam	28	62.29%
Vancomycin	18	43.90%
Meropenem	16	39.00%
Clindamycin	15	36.58%

**Table 8:** Tailored antibiotics

Antibiotic	Frequency	Percentage
Piperacillin/tazobactam	18	47.37%
Meropenem	18	47.37%
Clindamycin	11	28.20%
Vancomycin	10	28.95%

Postoperative care

Various approaches were employed in wound care for patients with Fournier gangrene. Vacuum-assisted closure (VAC) dressing was the most commonly used method in 63.41% of cases. Regarding skin cover, primary closure after adequate debridement was the most frequently employed method (56.1%, Table 9).

**Table 9:** Postoperative care

Parameter	Frequency	Percentage
Wound care		
VAC dressing	26	63.41%
Iodine dressing	15	36.58%
Skin cover		
Primary wound closure	23	56.10%
Secondary wound closure	10	24.39%
Skin graft	8	19.51%

## Discussion

Fournier gangrene, as described earlier, is a surgical emergency with rapid progression to sepsis and death with high mortality rates. The study showed that the total number of cases of Fournier gangrene at the National Guard Hospital was 41 between 2015 and 2022. The population of the study was mostly male, with a male-to-female ratio of 19:1. The diagnosis was made clinically and confirmed through imaging studies. Treatment was urgent surgical debridement coupled with broad-spectrum antibiotic administration, which was then tailored to the specific causing organism grown in the collected tissue culture. The mortality rate was as low as 15%. This can be attributed to the availability of prompt services in our hospital with a multidisciplinary team approach containing but not limited to urologic, general, and plastic surgeons, nursing staff, emergency department physicians, infectious disease specialists, and intensive care units. The local trends observed in this study showed some differences from similar studies conducted in other parts of the world. According to Barreda et al., they have found that alcoholism and immunosuppression were major risk factors; in contrast, we have found low rates of both risk factors in our population [8].

A study found that Fournier gangrene accounts for 0.02% of all United States hospital admissions. The incidence was found to be 1.6% per 100,000 patients in the male population, mostly in males aged 50-79 years old. Meanwhile, the female population has an incidence of 0.25% per 100,000 patients. Thus, Fournier gangrene has a significant predilection for males. However, females tend to present more acutely and require more interventions [9,10]. Unfortunately, the aggressive disease process has a high mortality rate of 20-40% [11,12].

A global study done in 2022 determined that the best treatment for Fournier gangrene includes fluid resuscitation followed by broad-spectrum antibiotics and extensive surgical debridement [13]. In Fournier gangrene, the infected area has a feculent stench and is large, dark, and coated in macerated skin with early systemic septic shock symptoms that are frequently present [14]. Another local study also shows that co-morbidities such as diabetes, cardiovascular disease, and kidney disease must be included in the FGSI as an important prognostic tool for Fournier gangrene mortality [15].

We have also found evident similarities in our patients to international data; for instance, tissue cultures revealed that the most common finding was mixed bacteria, followed by E. coli and Streptococci, as shown in Figure 5, which is consistent with international data as per Tang et al. [3]. The study is limited by the population size, which is low due to the rarity of the disease; however, it is relatively larger than similar studies. Also, it provides insight into the incidence and mortality rate in relation to demographic data of the disease in the local population, which gives good epidemiological data that can be used to draw strategies and policies for prevention at the local level. 

For future studies, we recommend including more hospitals to cover a larger patient population, which would allow for validation of the demographic trends and enable more robust inferential analyses.

## Conclusions

To conclude, this article studied Fournier gangrene, which is a surgical emergency that requires prompt attention with resuscitation, antibiotic therapy, and surgical debridement. It identified the demographic factors of patients who presented with the disease and provided the incidence rate, mortality rate, and outcomes of the disease. It also described the specifics of management and hospital courses. It has been found that patients who present to the National Guard Hospital with Fournier Gangrene are most likely to be males, diabetics, with a mean age of 60, and that the mortality rate is 15%.

## References

[1] Chernyadyev SA, Ufimtseva MA, Vishnevskaya IF (2018). Fournier’s gangrene: literature review and clinical cases [Internet]. Urol Int.

[2] Short B (2018). Fournier gangrene: an historical reappraisal. Intern Med J.

[3] Tang LM, Su YJ, Lai YC (2015). The evaluation of microbiology and prognosis of fournier's gangrene in past five years. Springerplus.

[4] Pastore AL, Palleschi G, Ripoli A (2013). A multistep approach to manage Fournier's gangrene in a patient with unknown type II diabetes: surgery, hyperbaric oxygen, and vacuum-assisted closure therapy: a case report. J Med Case Rep.

[5] Shyam DC, Rapsang AG (2013). Fournier's gangrene. Surgeon.

[6] Lewis GD, Majeed M, Olang CA, Patel A, Gorantla VR, Davis N, Gluschitz S (2021). Fournier’s gangrene diagnosis and treatment: a systematic review. Cureus.

[7] Syllaios A, Davakis S, Karydakis L (2020). Treatment of Fournier's gangrene with vacuum-assisted closure therapy as enhanced recovery treatment modality. In Vivo.

[8] Torremadé Barreda J, Millán Scheiding M, Suárez Fernández C, Cuadrado Campaña JM, Rodríguez Aguilera J, Franco Miranda E, Biondo S (2010). [Fournier gangrene. A retrospective study of 41 cases]. Cir Esp.

[9] Auerbach J, Bornstein K, Ramzy M, Cabrera J, Montrief T, Long B (2020). Fournier gangrene in the emergency department: diagnostic dilemmas, treatments and current perspectives. Open Access Emerg Med.

[10] Sorensen MD, Krieger JN (2016). Fournier’s gangrene: epidemiology and outcomes in the general US population. Urol Int.

[11] Singh A, Ahmed K, Aydin A, Khan MS, Dasgupta P (2016). Fournier's gangrene. A clinical review. Arch Ital Urol Androl.

[12] Leslie SW, Rad J, Foreman J (2023). Fournier Gangrene. https://www.ncbi.nlm.nih.gov/books/NBK549821/.

[13] Huayllani MT, Cheema AS, McGuire MJ, Janis JE (2022). Practical review of the current management of Fournier's gangrene [Internet]. Plast Reconstr Surg Glob Open.

[14] Katib A, Al-Adawi M, Dakkak B, Bakhsh A (2013). A three-year review of the management of Fournier's gangrene presented in a single Saudi Arabian institute. Cent European J Urol.

[15] El-Qushayri AE, Khalaf KM, Dahy A (2020). Fournier's gangrene mortality: a 17-year systematic review and meta-analysis. Int J Infect Dis.

